# Acute exposure to sublethal doses of neonicotinoid insecticides increases heat tolerance in honey bees

**DOI:** 10.1371/journal.pone.0240950

**Published:** 2022-02-25

**Authors:** Victor H. Gonzalez, John M. Hranitz, Mercedes B. McGonigle, Rachel E. Manweiler, Deborah R. Smith, John F. Barthell

**Affiliations:** 1 Undergraduate Biology Program and Department of Ecology and Evolutionary Biology, University of Kansas, Lawrence, Kansas, United States of America; 2 Biological and Allied Health Sciences, Bloomsburg University, Bloomsburg, Pennsylvania, United States of America; 3 Department of Ecology and Evolutionary Biology, University of Kansas, Lawrence, Kansas, United States of America; 4 Department of Biology, University of Central Oklahoma, Edmond, Oklahoma, United States of America; Institut Sophia Agrobiotech, FRANCE

## Abstract

The European honey bee, *Apis mellifera* L., is the single most valuable managed pollinator in the world. Poor colony health or unusually high colony losses of managed honey bees result from a myriad of stressors, which are more harmful in combination. Climate change is expected to accentuate the effects of these stressors, but the physiological and behavioral responses of honey bees to elevated temperatures while under simultaneous influence of one or more stressors remain largely unknown. Here we test the hypothesis that exposure to acute, sublethal doses of neonicotinoid insecticides reduce thermal tolerance in honey bees. We administered to bees oral doses of imidacloprid and acetamiprid at 1/5, 1/20, and 1/100 of LD_50_ and measured their heat tolerance 4 h post-feeding, using both dynamic and static protocols. Contrary to our expectations, acute exposure to sublethal doses of both insecticides resulted in higher thermal tolerance and greater survival rates of bees. Bees that ingested the higher doses of insecticides displayed a critical thermal maximum from 2 ˚C to 5 ˚C greater than that of the control group, and 67%–87% reduction in mortality. Our study suggests a resilience of honey bees to high temperatures when other stressors are present, which is consistent with studies in other insects. We discuss the implications of these results and hypothesize that this compensatory effect is likely due to induction of heat shock proteins by the insecticides, which provides temporary protection from elevated temperatures.

## Introduction

Animal pollination is essential for plant reproduction, ecosystem maintenance, and food security, as about 75% of the leading global food crops depend partially or fully on pollinators [1]. The single most valuable pollinator species in the world, found in both agricultural and natural habitats, is the European honey bee *Apis mellifera* L. [2]. In the U.S. alone, honey bees provide at least $15 billion worth of pollination services and generate from 300 to 500 million dollars in harvestable honey and other products each year [3]. However, managed honey bees are under pressure from myriad stressors that include habitat loss, parasites, diseases, pesticides, and poor nutrition. Bees are now exposed to multiple, simultaneous stressors throughout their lives, which has resulted in unusually high annual colony losses or significant declines in colony health [4].

Several studies demonstrate that the combined effects of stressors are more harmful to bees than one stressor alone [4]. For example, exposure to sublethal doses of neonicotinoid insecticides and nutritional stress renders honey bees more susceptible to the impact of the microsporidian parasite *Nosema* Nägueli, resulting in low brood and adult population sizes [5, 6]. In addition, stressors may act synergistically and thus cause significant harm to bees. For instance, hive mortality increases when bees are exposed to *Nosema* and to sublethal doses of neonicotinoids or nutritional stress, since the latter two stressors may suppress immunity [7, 8]. Thus, understanding the potential effects resulting from interactions among stressors is relevant for honey bee management and protection.

Climate change is a major new potential stressor altering global temperatures, rainfall, and wind patterns [9]. More severe and frequent extreme weather events are expected, and these will likely accentuate the effects of the stressors that honey bees already face. Alterations in temperature and rainfall are likely to cause spatial, temporal, morphological, and recognition mismatches between plants and pollinators [10]. Changes in the geographical distribution, development, and productivity of honey bees are anticipated, and some studies already document the negative effect of droughts on productivity and survival of honey bee colonies [11]. Climate change will also facilitate the spread of parasites and diseases or intensify their deleterious interactions with honey bees [12]. Clearly, assessing the physiological and behavioral responses of honey bees to high temperatures under the influence of one or more stressors is a priority.

Few studies have addressed honey bee thermal biology in the presence of other stressors, and the results are not encouraging. For example, acute exposures to diesel exhaust reduces heat tolerance in honey bees, which is concerning because air pollution continues to increase due to rising human population levels and agricultural intensification [13]. In addition to the direct mortality caused by pesticides, their sublethal effects can have significant impacts on bees’ physiology and behavior [14]. Given that insecticides become more toxic at higher temperatures, and that their use is expected to increase under global warming [15], we are therefore interested in determining if insecticides alter the heat tolerance of honey bees.

Here we assess the effect of acute sublethal doses (1/5, 1/20, and 1/100 of LD_50_) of imidacloprid and acetamiprid on honey bees’ thermal tolerance. We chose these two systemic neonicotinoid insecticides because they are widely used in agriculture for pest control, and they have been documented to cause detrimental effects on honey bees. Although imidacloprid is more toxic to honey bees than acetamiprid [16], sublethal doses of both insecticides similarly affect their physiology and behavior, such as learning and memory performance, homing ability, foraging, immunocompetence, and susceptibility to parasites [17–20]. To assess the effects of each insecticide on the heat tolerance of honey bees, we use dynamic (ramping temperatures) and static (constant temperature) protocols. In the dynamic protocol, we measure bees’ critical thermal maximum (CT_Max_) or the temperature at which an organism loses motor control [21]. Such a physiological parameter has a strong predictive power for understanding bees’ responses to changes in climate as well as land use [22–24]. In the static protocol, we measure bee survival after constant heat exposure. The results of this experiment will be informative about the effects of insecticides on bees’ ability to tolerate a heat stress event. Given the synergistic effects of neonicotinoids with other stressors [7, 8], we hypothesize that bees exposed to acute sublethal doses of insecticides will display a lower CT_Max_ and have a reduced rate of survival in comparison to individuals not exposed to insecticides.

## Materials and methods

We used honey bee foragers from an apiary located at the Native Medicinal Plant Research Garden (39˚00’37”N, 95˚12’23”W, 254 m) of the University of Kansas, Lawrence, Kansas, U.S.A. We conducted pilot studies with bees from a single Langstroth hive during the summer of 2020 and repeated experiments with bees from four additional hives during the summer of 2021. We trained bees to forage at a feeder containing a 1.5 M sucrose solution scented with either lavender or mint. For all assays, we captured foraging bees between 9:00 and 10:00 h with a glass vial at the feeder, which we then covered with a net mesh (1 mm in diameter). We kept bees inside a cooler (16–19 ˚C) until we completed fieldwork. Once in the laboratory, we immobilized bees in a refrigerator (3 ˚C) for 3–5 min and transferred them to 2 mL plastic vials, which had a small opening (2–3 mm in diameter) at one end and a net mesh on the other. Using a micropipette, we fed bees to satiation with 1.5 M sucrose solution through the vial’s opening or the net mesh. As in Hranitz et al. [25], we held bees overnight (21–22 h) at room temperature (21–22 ˚C) before experimentation to ensure all individuals had a similar motivation to feed.

### Insecticide doses

We used commercial formulations with imidacloprid (Macho^®^ 4.0, Agri Star^®^, Albaugh LLC, Ankeny, IA, USA) and acetamiprid (Ortho^®^, flower, fruit & vegetable insect killer, The Scotts Company LLC, Marysville, OH, USA) to prepare stock solutions of each pesticide. We used commercial formulations because we aimed to simulate field conditions by testing the products commonly applied by farmers. We used distilled water to prepare these stock solutions at a concentration of 407 ng/μL for imidacloprid and 500 ng/μL for acetamiprid. We diluted these stock solutions in 1.5 M sucrose to obtain the concentrations of insecticides used in the experiments. We used doses of each insecticide based on the LD_50_ value calculated from acute contact exposure from a topical application, 18 ng/bee for imidacloprid and 7100 ng/bee for acetamiprid [16]. We used the following doses for each insecticide: imidacloprid, 3.6 ng/bee (20% of the LD_50_), 0.9 ng/bee (5% of the LD_50_), and 0.18 ng/bee (1% of the LD_50_); acetamiprid, 1420 ng/bee (20% of the LD_50_), 355 ng/bee (5% of the LD_50_), and 71 ng/bee (1% of the LD_50_). Henceforth, the doses 20%, 5%, and 1% are referred as 1/5, 1/20, and 1/100 of LD_50_. As a control, we used 1.5 M sucrose solution without insecticide. These concentrations of pesticides did not induce mortality in the experimental population within the timeframe of the study. We kept all solutions refrigerated and prepared a new stock every week. We administered 10 μL of treatment solutions to bees orally, as previous studies showed that honey bees freely consumed solutions containing up to 40% of imidacloprid [19]. We measured bees’ CT_Max_ and survival after constant heat exposure at 4 h postfeeding, as previous studies indicated that this is the period in which both insecticides have the most effect on honey bees’ behavior (J. Hranitz, per. obs.).

### CT_Max_ assays

To measure CT_Max_, we followed Gonzalez et al. [26] in placing bees individually in sealed glass vials (7.4 ml; 17 × 60 mm) and submerging them horizontally (attached to a metal tray) at approximately 1 cm in depth within a water bath. We used a water bath with a volume of 12 L controlled by a thermostat (18–100 ˚C; Bellco Sci-Era Hot Shaker, Vineland, New Jersey). We used a dynamic ramping protocol with an initial temperature of 26 ˚C and held bees for 10 min before increasing it 1 ˚C every 2.5 min with an accuracy of ±0.1 ˚C. To estimate the temperature inside the tubes, we placed an iButton data logger (weight: 3.104 g; DS1923 Hygrochron™; Maxim Integrated, San Jose, California) inside a glass vial and submerged it in the water bath. Thus, we report the temperature inside the tubes not the temperature displayed by the thermostat of the water bath. Pilot experiments indicated that bees held in similar sealed glass vials adjacent to the water bath at room temperature survived through the duration of the CT_Max_ assays. Thus, observed bees’ responses inside sealed vials during our assays were due to changes in temperature, not to oxygen limitation. As an approximation of the CT_Max_, we used the temperature at which bees lost muscular control, spontaneously flipping over onto their dorsa and spasming [21, 27, 28]. We inspected and rotated each vial to determine if the bees had lost muscle control at every Celsius degree until all bees had reached their upper thermal limit.

### Acute heat stress event

To assess whether acute exposure to sublethal doses of insecticides affect the ability of honey bees to tolerate heat stress, we followed Reitmayer et al. [13] in exposing bees to 43 ˚C inside an incubator and monitored their survival every hour during 5 hours. We conducted this experiment during three consecutive days for each insecticide, collecting and feeding bees with the same doses as indicated above. We placed bees individually inside glass vials and plugged them with a moistened cotton ball (~ 0.2 mL of distilled water per cotton ball) to ensure enough humidity during the experiment. The response variable in this experiment was time to death.

### Data analyses

We conducted statistical analyses in R [29] and created boxplots and line graphs using GraphPad Prism version 7.04 (GraphPad Software, San Diego, CA, USA). We used a Linear Mixed-Effect Model (LMM) to assess effects of insecticide treatments on the CT_Max_. In this model, treatment served as a fixed factor while colony identity as a random factor. We implemented this model using the lme4 package [30] and assessed the significance of fixed effects using a Type II Wald χ2 test with the car package [31]. We used the lsmeans package [32] to conduct multiple pairwise comparisons with Bonferroni adjustment to assess for differences among groups. We used failure-time analyses to assess for differences in bee survival in the acute heat stress assays. We implemented a Cox proportional hazard model using the survival package [33], including treatment as a fixed factor and colony identity as a covariate, and conducting post hoc pairwise comparisons with a Log-rank test. To check for the proportional hazard assumption of each Cox model, we tested for independence between time and the corresponding set of scaled Schoenfeld residuals of each variable (treatment and colony identity) using the functions cox.zph in the survival package and ggcoxzph in the survminer package (S1 and S2 Figs; S4 Table).

## Results

**Fig 1:**
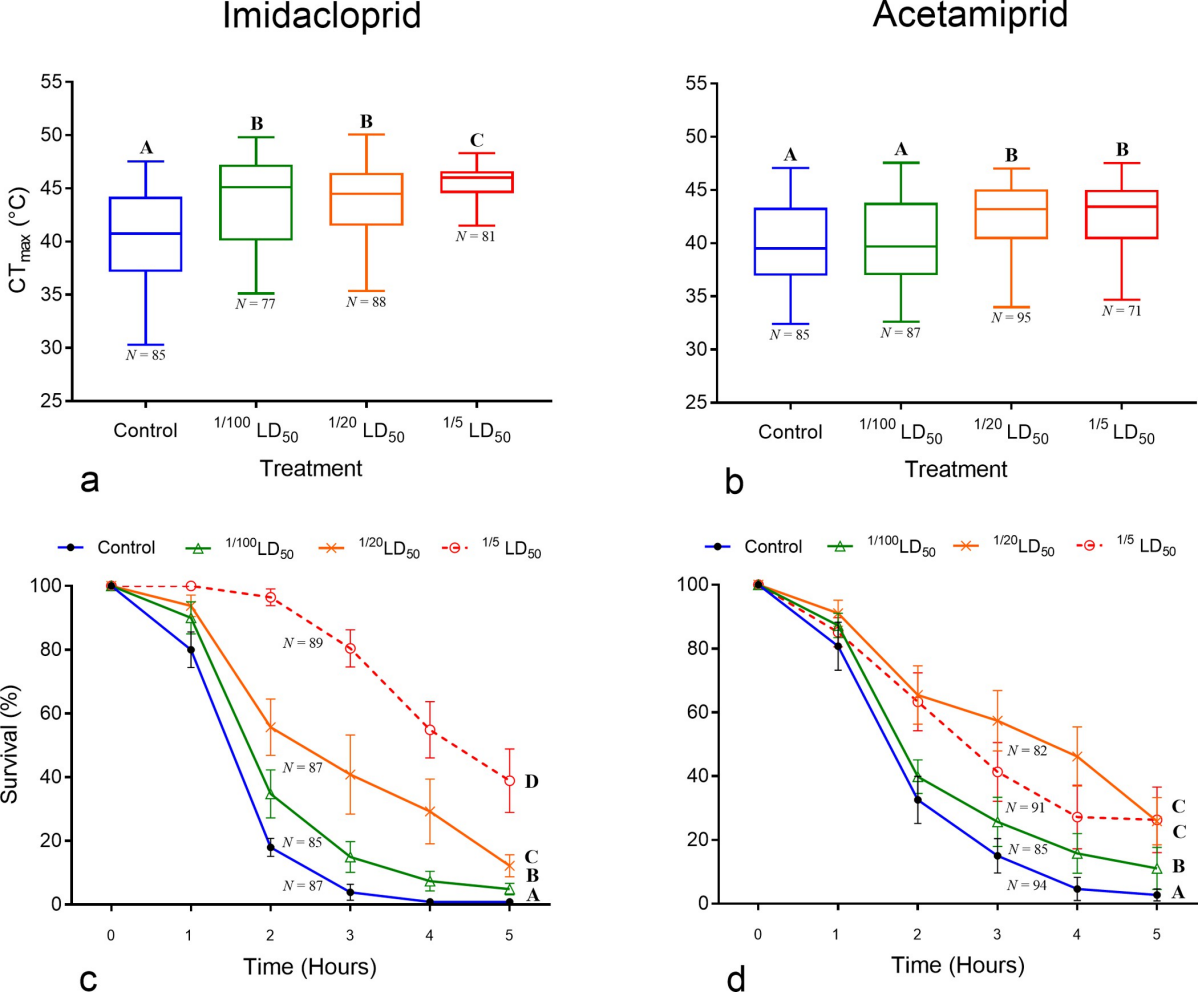
Effect of acute exposure to sublethal doses of neonicotinoid insecticides on the critical thermal maxima (CT_Max_) and survival of honey bee foragers. **a**, **b**, boxplots display median, quartiles, and extreme values of CT_Max_. **c**, **d**, bee survival (means ± SE) during a heat stress event (43 ˚C) over 5 hours. Different letters above boxplots and at the end of each survival curve indicate significant (*P <*0.05) mean differences.

The critical thermal maxima (CT_Max_) of honey bee foragers varied among treatments when we exposed them to acute sublethal doses of both insecticides (imidacloprid: Wald χ^2^ = 99.1; acetamiprid: Wald χ^2^ = 39.5, *DF* = 3 and *P <* 0.001 in both cases). Pairwise comparisons with Bonferroni adjustment detected differences in the CT_Max_ between the control group and all other bees treated with imidacloprid. The CT_Max_ of bees was similar among imidacloprid treatments, except for the highest dose (1/5 of LD_50_), and, on average, from 3.3 ˚C to 5.1 ˚C greater than that of the control group. We found a similar pattern in bees fed with acetamiprid, except that the CT_Max_ of bees fed with the lowest dose was like that of the control group. Bees fed with the two highest doses (1/20 and 1/5 of LD_50_) displayed a greater CT_Max_, on average, from 2.2 ˚C to 2.7 ˚C higher than the control group and bees fed with the lowest dose (see Fig 1A and 1B; S1 Table).

Bee survival also differed among treatments for both insecticides (imidacloprid: Wald χ^2^ = 153.6; acetamiprid: Wald χ^2^ = 78.6, *DF* = 7 and *P <* 0.001 in both cases). In general, survival rapidly decreased over time in bees of both the control group and those fed with the lowest dose (1/100 of LD_50_). However, bees fed with higher sublethal doses displayed greater survival rates. In comparison to the control group, hazard ratios (HR) indicated that mortality is reduced from 67% (HR: 0.33) in bees fed with 1/20 LD_50_ of acetamiprid, to 87% (HR: 0.13) in bees fed with 1/5 of LD_50_ of imidacloprid (Table 1). Pairwise comparisons with Bonferroni adjustment indicated differences in the survival of bees among all treatments with imidacloprid (S2 Table). For acetamiprid, bee survival was similar between the two higher doses, each one higher than the lowest dose (1/100 of LD_50_) and the control (see Fig 1C and 1D; S3 Table).

**Table 1:** Cox proportional hazards estimates of the survival of honey bees after exposure to three sublethal doses (LD_50_) of imidacloprid and acetamiprid.

	Imidacloprid		Acetamiprid	
Treatment	HR (95% CI)	*P*-value	HR (95% CI)	*P*-value
1/100 LD_50_	0.66 (0.49–0.90)	**0.009**	0.67 (0.49–0.90)	**0.009**
1/20 LD_50_	0.38 (0.28–0.52)	**<0.001**	0.33 (0.24–0.45)	**<0.001**
1/5 LD_50_	0.13 (0.09–0.19)	**<0.001**	0.44 (0.32–0.61)	**<0.001**

Survival measured following a heat stress event (43 ˚C) over 5 hours. *P*-values refer to comparisons with the control treatment. Significant values in boldface.

^HR^Hazard ratio

^CI^Confidence interval.

## Discussion

The deleterious effects on the development, behavior, physiology, and survival of honey bees due to acute and chronic exposures to sublethal doses of neonicotinoid insecticides, including imidacloprid and acetamiprid, have been widely documented in the literature [14, 20, 34–37]. Similarly, the synergistic adverse effects of insecticides with other stressors, such as poor nutrition and parasites, have been demonstrated [5–8]. Contrary to our expectations, acute exposure to sublethal doses of imidacloprid and acetamiprid had a positive effect on both honey bees’ CT_Max_ and survival following a heat stress event (43 ˚C). Bees fed with the higher doses of pesticides (1/20 and 1/5 of LD_50_) displayed a CT_Max_ from 2 ˚C to 5 ˚C greater than that of the control group and 67%–87% reduction in mortality (Fig 1, Table 1). Thus, these results do not support the hypothesis that acute, sublethal doses of neonicotinoid insecticides reduce heat tolerance in honey bees.

While unanticipated, our results are consistent with studies in other insect species. For example, Zhang et al. [38] indicate that a pesticide *non-resistant* strain of diamondback moth, *Plutella xylostella* (L.), is more thermotolerant than a resistant strain. As noted by these authors, the greater susceptibility to higher temperatures in the resistant strain likely relates to weaker uploading of heat shock proteins (HSP), among other factors. Heat shock proteins are chaperones that prevent the denaturing of other proteins under heat, as well as under other forms of stress such as cold, starvation, bacterial infections, and exposure to chemicals including pesticides [25, 39]. Inducible heat shock proteins in the HSP70 family of genes are variable in their expression within species, as in the case of the diamondback moth [40]. Similarly, in larval mosquitoes, induced cross-tolerance to a pesticide has been documented through preconditioning at high but sublethal temperatures [41]. Indeed, in honey bees, Koo et al. [42] indicate that heat shock protein expression varies with the type of stressor (including from heat shock), suggesting that pesticides may induce specific responses to various chemical exposures. Thus, we hypothesize that sublethal doses of insecticides activate a stress response in honey bees, which confers further stress resistance to high temperatures. Future studies will attempt to identify this expression profile in correlation with the pesticides used in this work.

The increase in CT_Max_ and greater survival of honey bees after exposing them to sublethal doses of neonicotinoids do not imply any potential benefits to honey bees’ thermal tolerance nor to their resistance to global warming. Instead, our results demonstrate the short-term resilience of honey bees to high temperatures when other stressors are present. The adverse effects on the behavior and physiology of honey bee’s foragers due to neonicotinoid insecticides are unquestionable, including for insecticides with low toxicity, such as acetamiprid, that have been promoted as a “bee-friendly” pesticide in the market. For example, both acute and chronic sublethal doses of imidacloprid adversely affect aversive learning and reduce overall daily activity, number of foraging trips, and overall lifespan of honey bee foragers [19, 43]. Similarly, sublethal doses of acetamiprid affect locomotor activity, sucrose sensitivity, and memory of honey bees [34]. Thus, although honey bee foragers exposed to acute sublethal doses of insecticides may survive high temperatures, they are behaviorally and physiologically impaired, which in the long-term will alter colony development and productivity.

Acute sublethal doses of pesticides also alter honey bees’ thoracic muscle activity, which allows bees to warm up by shivering their muscles (thermogenesis) and move their wings during flight and fanning the brood. Acute oral exposure to the neonicotinoid thiamethoxam impairs thermogenesis in African honey bees from one hour after exposure and for at least one day, which may not only affect their foraging activity but also other tasks within the colony, such as nest thermoregulation [44]. Similar disruptions to the thermogenic capacity of bees following acute and chronic exposures to both imidacloprid and acetamiprid have been documented in bumble bees [45, 46] and solitary bees [47]. At least under simulated heat wave events, honey bees increase water collection and brood ventilation by recruiting foragers [48]. Because these behaviors require bees to use their thoracic muscles, foragers under the influence of pesticides may be unable to accomplish these tasks successfully, which will influence nest homeostasis.

Honey bee foragers are exposed to pesticides through oral and contact exposures via contaminated nectar, pollen, and/or water [4, 47, 49–52]. Because of pesticide persistence in the environment, bees are exposed for long periods, not to one, but to a diverse array of pesticides as well as to other agrochemicals that include fungicides and herbicides [49, 53]. However, recent studies demonstrate that exposure to multiple compounds result in synergistic effects, which often increase the toxicity of individual pesticides, although levels of synergism among pesticides depend on the residue levels, ratio of pesticides, and their mode of action [54]. For instance, acetamiprid becomes more toxic when combined with triazole fungicides because the latter may inhibit P450-mediated detoxification [49]. Among 98 binary to octonary mixtures of acetamiprid in combination with seven pesticides, 45% of them exhibited synergistic effects on honey bees [55]. Similarly, deltamethrin induces hypothermia on honey bees when combined with the azole fungicide prochloraz, but not when used alone [14, 56]. Because we used acute sublethal doses of individual pesticides in our laboratory experiments, we do not know if bees would display similar responses to a combination of pesticides and to chronic exposures. It is likely that cumulative toxicity due to a chronic exposure, as well as an increase in toxicity by a combination of pesticides, would inhibit the stress protein response, thus resulting in a lower heat tolerance. Doubtless, future studies should address both factors (combination of agrochemicals and chronic exposures) to obtain a more realistic view of the effects of pesticides on honey bee thermal biology. Similarly, future studies should assess for potential synergistic effects of multiple stressors on the bees’ thermal biology, such as the combined effects of pesticides with nutritional stress or parasites. To date, only one study has addressed these effects in a species of dung beetle exposed to both ivermectin, a toxic parasiticide, and an immune challenge [57]. The authors found no apparent additive or synergistic effects between these two stressors, as heat tolerance increased only in immune-challenged beetles but not in those exposed to ivermectin. Thus, this interesting study indicates that multiple stressors not always induce additive or synergistic effects, and that responses are specific to each type of stressor.

Although we tested bees collected from a feeder to select foragers, we were unable to control for their age. Several studies have documented a negative relationship between age and heat tolerance in many insects [58, 59], including bumble bees [60]. Thus, the thermal tolerance of honey bees as well as their response to pesticides may vary depending on age. A mixture of bees from different ages could also explain the high variation in the CT_Max_ observed in our experiments, which ranged from 32 ˚C to 47 ˚C across treatments (Fig 1). In addition, we measured CT_Max_ as the temperature at which a bee lost muscular control using a dynamic protocol, which requires the visual detection of this physiological event [21]. Detecting this physiological endpoint was particularly challenging in bees that ingested the highest doses of insecticides, which were clearly lethargic from the beginning of the experiment. We are confident with our measurements of CT_Max_ because they are congruent with the results obtained using the static protocol. However, using thermolimit respirometry may be a better approach in these cases, as that method provides a more accurate measurement of CT_Max_ by combining metabolic rate (V_CO2_) and motor activity [61].

To our knowledge, this work is the first in documenting the effects of sublethal doses of pesticides on the heat tolerance of any bee species. Although our results appear counterintuitive at first, they are consistent with results from experiments in other insect species addressing similar questions [38, 40, 41]. Stimulatory responses to low doses of pesticides have been documented in some arthropods, particularly in pest species, but these effects often remain unnoticed or unappreciated [62, 63]. As a post hoc hypothesis, we suggest that sublethal doses of insecticides induce the expression of HSPs, which confers further stress resistance to high temperatures. Despite the essential role of temperature and humidity in the development, survival, and health of honey bee colonies [64], as well as concerns about the impact of climate change on pollinators and pollination, it is surprising that the effects of environmental stressors on the bees’ thermal biology have been largely overlooked.

## Supporting information

S1 Fig Distribution of the scaled Schoenfeld residuals against the transformed time for each variable (treatment and date) of the Cox model built to assess the survival of honey bees after exposure to acute sublethal doses of imidacloprid followed by a heat stress event (43 ˚C) over 5 hours. (DOCX)

S2 Fig Distribution of the scaled Schoenfeld residuals against the transformed time for each variable (treatment and date) of the Cox model built to assess the survival of honey bees after exposure to acute sublethal doses of acetamiprid followed by a heat stress event (43 ˚C) over 5 hours. (DOCX)

S1 Table *P*-values of pairwise comparisons with Bonferroni adjustment of the critical thermal maxima (CT_Max_) displayed by honey bee foragers after acute exposure to sublethal doses of imidacloprid and acetamiprid. *DF* = 1 in all comparisons. Significant *P*-value in boldface.
(DOCX)

S2 Table *P*-values of pairwise comparisons of honey bee survival using a Log-rank test after acute exposure to sublethal doses of imidacloprid followed by a heat stress event (43 ˚C) over 5 hours. Significant *P*-value in boldface.
(DOCX)

S3 Table *P*-values of pairwise comparisons of honey bee survival using a Log-rank test after acute exposure to sublethal doses of acetamiprid followed by a heat stress event (43 ˚C) over 5 hours. Significant *P*-value in boldface.
(DOCX)

S4 Table Results of test for independence between time and the corresponding set of scaled Schoenfeld residuals of each variable (treatment and date) used in a Cox proportional hazard model. This model was used to assess for survival of honey bees after exposure to acute sublethal doses of neonicotinoid insecticides (imidacloprid and acetamiprid) followed by a heat stress event (43 ˚C) over 5 hours. DF = degrees of freedom.
(DOCX)
