# Supplementary material for: Acute exposure to sublethal doses of neonicotinoid insecticides increases heat tolerance in honey bees
Source: PLoS One. 2022 Feb 25;17(2):e0240950. doi: 10.1371/journal.pone.0240950 (PMC8880832; doi:10.1371/journal.pone.0240950)

**S1 Figure.** Distribution of the scaled Schoenfeld residuals against the transformed time for each variable (treatment and date) of the Cox model built to assess the survival of honey bees after exposure to acute sublethal doses of imidacloprid followed by a heat stress event (43 ˚C) over 5 hours.


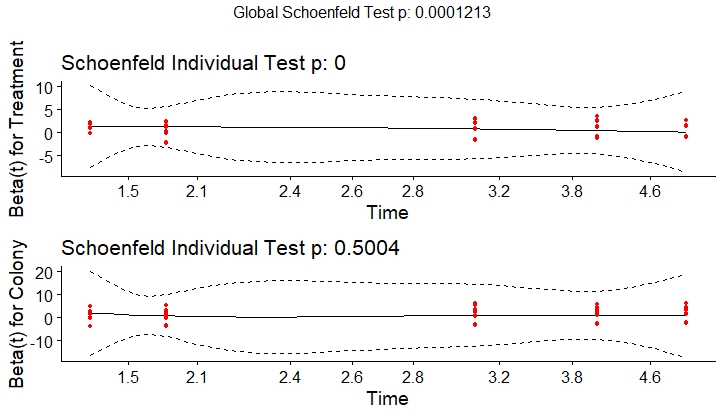

Supplement: S1 Fig — (DOCX) [file pone.0240950.s001.docx]
