# Supplementary material for: Acute exposure to sublethal doses of neonicotinoid insecticides increases heat tolerance in honey bees
Source: PLoS One. 2022 Feb 25;17(2):e0240950. doi: 10.1371/journal.pone.0240950 (PMC8880832; doi:10.1371/journal.pone.0240950)
Supplement: S1 Table — DF = 1 in all comparisons. Significant P-value in boldface. (DOCX) [file pone.0240950.s003.docx]

**S1 Table.** *P*-values of pairwise comparisons with Bonferroni adjustment of the critical thermal maxima (CT_Max_) displayed by honey bee foragers after acute exposure to sublethal doses of imidacloprid and acetamiprid. *DF* = 1 in all comparisons. Significant *P*-value in boldface.

| **Comparison** | **Pesticide** | |
| --- | --- | --- |
| Treatments | Imidacloprid | Acetamiprid |
| Control – 1/100 LD_50_ | **< 0.0001** | 1.000 |
| Control – 1/20 LD_50_ | **< 0.0001** | **< 0.0001** |
| Control – 1/5 LD_50_ | **< 0.0001** | **0.0001** |
| 1/100 LD_50_ – 1/20 LD_50_ | 1.000 | **0.0002** |
| 1/100 LD_50_ – 1/5 LD_50_ | **0.009** | **0.0010** |
| 1/20 LD_50_ – 1/5 LD_50_ | **0.008** | 1.000 |
