# Supplementary material for: Acute exposure to sublethal doses of neonicotinoid insecticides increases heat tolerance in honey bees
Source: PLoS One. 2022 Feb 25;17(2):e0240950. doi: 10.1371/journal.pone.0240950 (PMC8880832; doi:10.1371/journal.pone.0240950)
Supplement: S2 Table — Significant P-value in boldface. (DOCX) [file pone.0240950.s004.docx]

**S2 Table.** *P*-values of pairwise comparisons of honey bee survival using a Log-rank test after acute exposure to sublethal doses of imidacloprid followed by a heat stress event (43 ˚C) over 5 hours. Significant *P*-value in boldface.

|  | Control | 1/100 LD_50_ | 1/20 LD_50_ | 1/5 LD_50_ |
| --- | --- | --- | --- | --- |
| Control | – | **0.013** | **<0.0001** | **<0.0001** |
| 1/100 LD_50_ | **0.013** | – | **0.002** | **<0.0001** |
| 1/20 LD_50_ | **<0.0001** | **0.002** | – | **<0.0001** |
| 1/5 LD_50_ | **<0.0001** | **<0.0001** | **<0.0001** | – |
