# Supplementary material for: Acute exposure to sublethal doses of neonicotinoid insecticides increases heat tolerance in honey bees
Source: PLoS One. 2022 Feb 25;17(2):e0240950. doi: 10.1371/journal.pone.0240950 (PMC8880832; doi:10.1371/journal.pone.0240950)
Supplement: S4 Table — This model was used to assess for survival of honey bees after exposure to acute sublethal doses of neonicotinoid insecticides (imidacloprid and acetamiprid) followed by a heat stress event (43 ˚C) over 5 hours. DF = degrees of freedom. (DOCX) [file pone.0240950.s006.docx]

**S4 Table.** Results of test for independence between time and the corresponding set of scaled Schoenfeld residuals of each variable (treatment and date) used in a Cox proportional hazard model. This model was used to assess for survival of honey bees after exposure to acute sublethal doses of neonicotinoid insecticides (imidacloprid and acetamiprid) followed by a heat stress event (43 ˚C) over 5 hours. DF = degrees of freedom.

|  |  | Imidacloprid | | Acetamiprid | |
| --- | --- | --- | --- | --- | --- |
| Variable | DF | *X^2^* | *P*-value | *X^2^* | *P*-value |
| Treatment | 3 | 26.31 | < 0.0001 | 0.40 | 0.94 |
| Colony | 4 | 3.35 | 0.5 | 2.27 | 0.69 |
| Global | 7 | 29.42 | 0.0001 | 2.62 | 0.92 |
